# Study on the care burden and influencing factors of family caregivers for demented older people in China

**DOI:** 10.1002/alz.083877

**Published:** 2025-01-09

**Authors:** Ning Sun

**Affiliations:** ^1^ Ningbo College of Health Sciences, Ningbo, zhejiang China

## Abstract

**Background:**

To understand the current situation of the care burden of elderly caregivers in the community and explore the influencing factors.

**Method:**

100 family caregivers in community were assessed using the General information questionnaire and Home Caregiver Burden Scale (CBI) in Ningbo City In China.

**Results:**

The care burden’s average scores of 100 family caregivers in community was 52.36±12.08, with a score of 58.10%. Multiple regression analysis showed that the factors affecting the care burden included the severity of dementia in the elderly, whether there were accidents in the past year and the monthly income of the family (*P*<0.05, *P*<0.01).

**Conclusion:**

There is a certain degree of care burden for the family caregivers of the demented elderly in the community. It is necessary to strengthen the concern for the family caregivers of the demented elderly in the community, and the guidance of the dementia nursing intervention and the self‐life intervention guidance of the caregiver in the community. The burden of care can be reduced by improving their care capacity.

